# Control Framework for Sloped Walking With a Powered Transfemoral Prosthesis

**DOI:** 10.3389/fnbot.2021.790060

**Published:** 2022-01-11

**Authors:** Namita Anil Kumar, Shawanee Patrick, Woolim Hong, Pilwon Hur

**Affiliations:** ^1^Department of Mechanical Engineering, Texas A&M University, College Station, TX, United States; ^2^Department of Mechanical Engineering, Gwangju Institute of Science and Technology, Gwangju, South Korea

**Keywords:** transfemoral prosthesis control, impedance control, rehabilitation, sloped walking, biomedical

## Abstract

User customization of a lower-limb powered Prosthesis controller remains a challenge to this date. Controllers adopting impedance control strategies mandate tedious tuning for every joint, terrain condition, and user. Moreover, no relationship is known to exist between the joint control parameters and the slope condition. We present a control framework composed of impedance control and trajectory tracking, with the transitioning between the two strategies facilitated by Bezier curves. The impedance (stiffness and damping) functions vary as polynomials during the stance phase for both the knee and ankle. These functions were derived through least squares optimization with healthy human sloped walking data. The functions derived for each slope condition were simplified using principal component analysis. The weights of the resulting basis functions were found to obey monotonic trends within upslope and downslope walking, proving the existence of a relationship between the joint parameter functions and the slope angle. Using these trends, one can now design a controller for any given slope angle. Amputee and able-bodied walking trials with a powered transfemoral prosthesis revealed the controller to generate a healthy human gait. The observed kinematic and kinetic trends with the slope angle were similar to those found in healthy walking.

## 1. Introduction

Despite decades of research in the field of human rehabilitation, energetically passive devices are the only commercially available solutions to a population of 1.3 million lower-limb amputees (Ziegler-Graham et al., 2008). An energetically passive device is one that stores and dissipates energy without providing net positive work to the gait cycle. The lacking positive work is compensated for by the user's residual limb, which overexerts the hip and pelvic muscles, eventually leading to severe gait asymmetries (Kaufman et al., 2012). Powered prostheses, on the other hand, provide a net positive work and consequently lower a user's metabolic cost (Herr and Grabowski, 2012; Goldfarb, 2013). The Ossur Power knee is the only powered prosthesis currently on the market, however it tends to not fair well with middle aged and older users (Hafner and Askew, 2015). It also performs poorly while walking on sloped terrain (Wolf et al., 2012; Morgenroth et al., 2018). Other Powered prosthesis knees remain viable only in academic settings due to numerous challenges. Setting aside the more obvious challenges like battery limitations and the bulkiness of motors, a less tackled obstacle is the difficulty in customizing the powered prosthesis to the user.

User customization of a prosthesis involves changes to the mechanical and control system. Mechanical customization is actively studied and some solutions include customized sockets, adjustable height and foot stiffness (Colombo et al., 2010; Fey et al., 2013; Comotti et al., 2015; Beck et al., 2017; Lecomte et al., 2021). Customization of control systems, on the contrary, has seen minor contributions with the most significant being the implementation of machine learning for auto-tuning level walking control parameters (Wen et al., 2020). The lack of contributions on this topic is primarily due to the problem's sheer magnitude. Since each mode of operation (e.g., standing, walking, stair ascent or descent) has its own control law, user customization of the control system involves tuning an unmanageable large number of tuning parameters. At this point, any solution that simplifies this behemoth of a task is appreciated. In this paper, we will focus on walking controllers for transfemoral prostheses on sloped terrain. We will study the complexities of walking on slopes and then propose a framework with far fewer tuning parameters than the state-of-the-art, thus simplifying user-customization of prosthesis control.

### 1.1. Background on Sloped Walking Control

**Figure 1:**
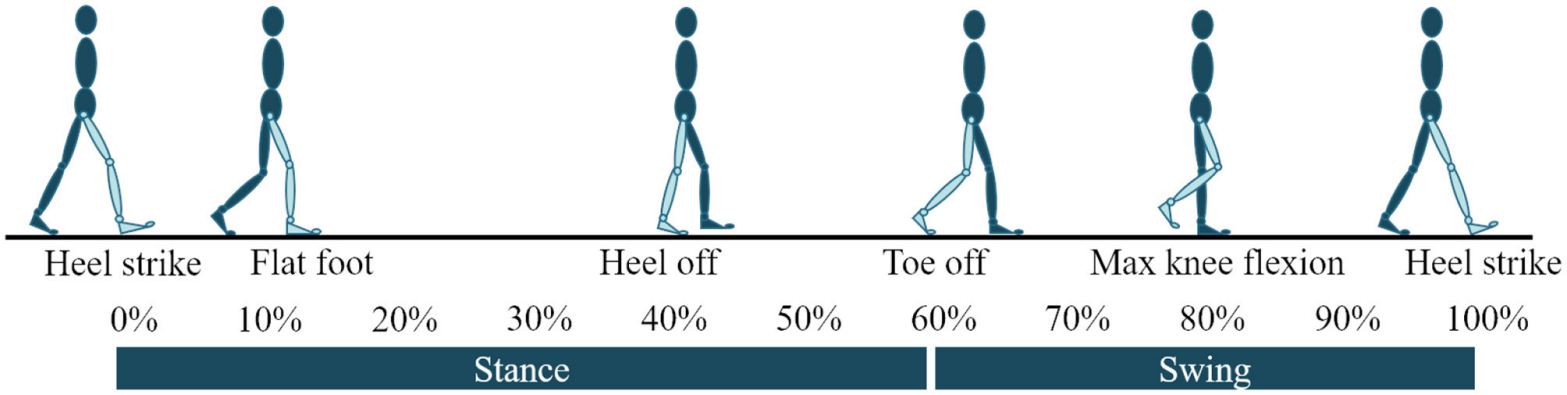
Gait cycle with important kinematic moments used as switching conditions in a finite state machine.

There are two well-known approaches to prosthesis walking control: impedance control and variants of feedback linearization. The known implementations of the latter are limited to level and upslope walking (Paredes et al., 2016). The former has been extensively used for level and sloped walking (both upslope and downslope). Almost all implementations of impedance control involves sectioning a gait cycle into 4–6 phases. These phases form the states in a finite state machine. A gait cycle is defined to begin and end with a heel-strike on the same limb. We will refer to the progress in a gait cycle using *t* which is 0 at gait cycle initiation and 1 (equivalent to 100%) at completion. Important kinematic moments in the gait cycle like heel-off and maximum knee flexion during swing phase are chosen as switching points between states. Figure 1 presents the gait cycle with important kinematic instances. The control input at any instant *t* is given by


(1) $$\begin{array}{l} \tau \left(t\right)=K\left(\theta \left(t\right)-\theta_{ref}\right)+D\dot{\theta }\left(t\right) \end{array}$$

where *K* and *D* represent the joint stiffness and viscous damping, respectively. The term θ_*ref*_ is the reference or equilibrium angle of the joint, while θ(·) and $\dot{\theta }\left(\cdot \right)$ signify the joint's position and velocity.

Within each state of the finite state machine, the joint parameters (i.e., *K*, *D*, and θ_*ref*_) can be assigned constant values or vary as a function of some gait characteristic. In Sup et al. (2008), the joint parameters were constant within each state in the finite state machine. Estimates for the parameters were determined through a least squares optimization that minimized the difference between the torque from Equation (1) and the joint torque from healthy human walking data. While this approach has been proven to emulate healthy walking kinematics and kinetics, it involves careful tuning of the initially estimated joint parameters (numbering at 12–18 per joint). In Sup et al. (2011), the authors recognized similarities between gait kinematics and kinetics on different slope angles, and suggested using the same impedance control strategy as in Sup et al. (2008) but with different joint parameters. Despite its success, this process involved re-tuning the joint parameters for every slope angle. Wen et al. (2020) attempted solving this issue through machine learning, but their attempts are limited to level walking. Additionally, the manner in which we produce a labeled data-set is debatable since we are yet to quantify crucial parameters like user comfort.

Varying the parameters as a function of gait characteristics has the benefit of fewer states in the finite state machine and hence fewer tuning parameters. Fey et al. (2014) and Bhakta et al. (2019) varied *K* and θ_*ref*_ as functions of the joint angle and the vertical ground reaction force during mid and terminal stance phases. The parameters were held constant during all other states in the finite state machine. While amputee trials proved the controller's success, the results in Fey et al. (2014) were limited to level and upslope walking and Bhakta et al. (2019) did not discuss gait kinetics. Furthermore, the controller's reliance on a load cell increases the ultimate cost and weight of the prosthesis. In Anil Kumar et al. (2020), the joint parameters varied as a function of *t* during stance phase, thus no longer requiring a load cell. However, the proposed control scheme was limited to the ankle joint and level walking. While the above approaches lessened the number of states during the stance phase, Lawson et al. (2014) and Hong et al. (2019) lessened the number of states during the swing phase by tracking healthy human walking trajectories. In fact, Hong et al. (2019) exploited the similarities between the sloped walking knee swing trajectories by tracking the level walking trajectory regardless of the slope angle. The smooth transitioning between stance and swing phases was facilitated by Bezier curves and a low gain PD controller toward the end of the gait cycle helped with terrain adaptation. Despite having fewer tuning parameters, the application of the above approaches to sloped walking still requires re-tuning several parameters for every slope angle.

### 1.2. Objectives

The problem of re-tuning the joint parameters for every slope angle is worsened by the absent relationship between the joint parameters and the slope angle. Our primary objective is to fill this gap in knowledge. The methods used in Anil Kumar et al. (2020) and Hong et al. (2019) form the foundation of our work. We first study the kinematics and kinetics of sloped walking, based on which we determine the objectives of our control framework for sloped walking (refer to section 2). In section 3, we present the control framework with our estimates of the joint control parameters across all slope angles. The estimation is an extension of the one presented in Anil Kumar et al. (2020) wherein *K* and *D* are polynomials of *t*. Upon estimating the joint control parameters for all slope angles, we extract basis functions spanning the entire set and propose a mapping between the joint parameters and the slope angle. Said mapping and the basis functions form the two contributions of this paper. In section 4, we discuss the implementation of our control framework on a powered transfemoral prosthesis. We also present a thorough tuning regime for our control strategy. The experimental results with an amputee and an able-bodied subject are then reported and discussed in section 5. Section 6 will have our concluding remarks.

## 2. A Brief Analysis of Sloped Walking

General practice in the field of walking assistive devices deems a device successful if it can emulate healthy gait kinetics and kinematics. In accordance to this norm, we determined control objectives by studying sloped walking kinematics and kinetics. Useful resources include: a *n* = 20 study by Montgomery and Grabowski (2018), a *n* = 10 study by Embry et al. (2018b) which also has a publicly available data-set (Embry et al., 2018a). The study (Montgomery and Grabowski, 2018) presents data for 7 slope angles (−9° to +9° at 3° increments), while the study (Embry et al., 2018a) presents results for 9 slope angles (−10° to +10° at 2.5° increments). We discuss the kinematics and kinetics of sloped walking in the following sections. Since our goal is to design a controller for a transfemoral prosthesis, we limit our discussion to the knee and ankle joint. The highlighted points will form the means by which we evaluate the performance of our controller.

### 2.1. Kinematics

Some important kinematic aspects of sloped walking are as follows. (i) The switching conditions of a finite state machine (shown in Figure 1) change with the slope and walking speed. The instants of flat-foot (ϕ_*FF*_) and heel-off (ϕ_*HO*_) occur earlier as the slope angle varies from steep downslope to steep upslope. On the other hand, toe-off (ϕ_*TO*_) is delayed as the slope varies. (ii) The amount of ankle plantar-flexion at toe-off increases as the slope varies from steep downslope to steep upslope. (iii) The ankle angle at the beginning of the gait cycle changes with the slope angle to facilitate terrain adaptation (i.e., the ankle is more dorsiflexed on upslopes). (iv) The amount of knee-flexion during initial stance phase increases with the steepness of the slope be it upslope or downslope.

### 2.2. Kinetics

The most important trends in sloped walking kinetics are: (i) the increase in push-off peak ankle torque and power as the slope varies from steep downslope to upslope; (ii) more knee flexion torque during initial stance phase on steeper slopes; (iii) more knee extension torque during terminal stance phase on upslopes. These trends are more strictly obeyed in Montgomery and Grabowski (2018), while the data pertaining to −5°, −2.5° in Embry et al. (2018a) deviate from the trends. In fact, the entire downslope walking torque data from Embry et al. (2018a) is higher than that found in Montgomery and Grabowski (2018) by a factor of 1.3–1.5. We believe (Montgomery and Grabowski, 2018) to be more accurate owing to the larger sample size. On the other hand, the data in Embry et al. (2018a) spans more slope conditions which helps greatly while determining the relationship between control parameters and the slope angle. So, we continue to use the data from Embry et al. (2018a), keeping in mind some anomalies are to be expected during downslope walking. We will account for these anomalies during implementation and accordingly adjust our final proposed control scheme.

## 3. Proposed Control Framework

As stated in Lawson et al. (2014), it is beneficial to use impedance control during stance phase since the limb is in contact with the terrain. During swing phase, it suffices to merely track healthy human trajectories. We thus propose a finite state machine with 4 states for the ankle and 5 for the knee. Both joints have three states during stance phase with the switches at ϕ_*FF*_, ϕ_*HO*_, and ϕ_*TO*_. In other words, State 1 begins at heel-strike and ends with ϕ_*FF*_, followed by State 2 which concludes at ϕ_*HO*_. State 3, the last state in the stance phase, ends at ϕ_*TO*_. During these three states, we adopted the same strategy as in Anil Kumar et al. (2020). That is, *K* and *D* vary as polynomial functions of *t*, while θ_*ref*_ assumes constant values during each state.

During swing phase, ankle angle does not vary much regardless of the slope angle–a motion achievable using constant *K*, *D*, and θ_*ref*_ values. The knee, on the contrary, is more animated, requiring a more motion rich trajectory. To achieve the desired motion while having few tuning parameters, we adopted the strategy proposed in Hong et al. (2019) to control the knee joint. That is, a single level-walking trajectory is tracked using a PD controller regardless of the slope angle. The level walking trajectory in Embry et al. (2018a) was used as the desired trajectory. A Bezier curve was generated in real-time to smoothly transition from the instantaneous position and velocity at ϕ_*TO*_ to a predefined point in the level-walking desired swing trajectory. Refer to Supplementary Figure 1 for a pictorial representation of the control framework.

### 3.1. Estimation of Joint Parameter Functions

To emulate healthy human gait kinetics using the impedance control strategy, we select joints parameters such that the torque produced is similar to that of healthy human walking, say τ_*data*_. This study used the sloped walking data reported in Embry et al. (2018a) for τ_*data*_, θ, and $\dot{\theta }$. The latter two are replaced by real-time angle and velocity feedback during implementation. We formulate an optimization that minimizes the norm of the difference between τ in (Equation 1) and τ_*data*_. Since the knee is controlled via impedance control only during stance phase, the knee's impedance estimation (and thereby cost function) was limited to the stance phase.

Supposing *m* and *n* represent the order of the *K* and *D* polynomials, respectively, the impedance parameters at instant *t* ∈ [0, 1] can be computed as follows,


(2) $$K(t)=\{\begin{array}{ll} \sum_{i=0}^{m}k_{i}t^{i} & \text{for }0\leq t<\phi_{TO}\\ k_{0} & \text{for }\phi_{TO}\leq t\leq 1 \end{array}$$

(3) $$D(t)=\{\begin{array}{ll} \sum_{i=0}^{n}d_{i}t^{i} & \text{for }0\leq t<\phi_{TO}\\ d_{0} & \text{for }\phi_{TO}\leq t\leq 1 \end{array}$$

The coefficients of the stiffness and damping polynomials are given by *k*_*i*_ and *d*_*i*_, respectively. The stiffness and damping parameters are assigned the values *k*_0_ and *d*_0_ during the swing phase. Doing so enforces continuity of the impedance parameters at heel-strike [i.e., *K*(0) = *K*(1) and *D*(0) = *D*(1)]. Presented below is the optimization problem:


(4) $$\begin{array}{ll} \underset{\theta_{ref},k_{i},d_{i}}{\min}\; & ∥\tau_{data}-\tau {∥}_{2} \end{array}$$

(5) $$\begin{array}{ll} \text{Subject to: } & K\left(t\right)\geq 0\;D\left(t\right)\geq 0 \end{array}$$

(6) $$\begin{array}{l} \text{Continuity of }K\text{ and }D\text{ at }t=\phi_{TO} \end{array}$$

(7) $$\begin{array}{l} |\theta_{ref}|\leq c_{1} \end{array}$$

(8) $$\begin{array}{l} |\Delta \tau /\Delta t|\leq c_{2} \end{array}$$

The decision variables are {θ_*ref*_, *k*_*i*_, *d*_*i*_}, where θ_*ref*_ is a set of reference angles, one for each state of the finite state machine. The constraints listed in Equation (5) force *K* and *D* to be positive. The constraint Equation (8) assures continuity of the joint parameter functions at toe-off. The scalar, *c*_1_, is a bound on the reference angles. $c_{1}=16^{˙}$ for the ankle and $c_{1}=36^{˙}$ for the knee. Further, the constraint Equation (8) forces the resulting τ to be Lipschitz continuous with constant *c*_2_. Additional bounds were added, as needed, to restrict the value of the damping parameters. The optimization problem was solved using Scipy's minimization function. Owing to the non-convex nature of the problem, a unique solution does not exist. Results from perturbation studies (Lee et al., 2016) and past studies using least squares approaches (Sup et al., 2011) helped judge the feasibility of the estimated joint parameter functions. Future efforts will involve solving the optimization problem using heuristics to decouple the stiffness and reference angles, and guarantee convergence.

### 3.2. Joint Control Parameter Functions

For both the ankle and the knee, *m* = *n* = 4 achieved the best results. The resulting ankle control parameter functions obeyed some monotonic trends across slope angles: (A1) Ankle stiffness during State 1-2 (ϕ_*HS*_ to ϕ_*HO*_) was higher on steeper downslope and upslope terrain. The higher stiffness aids in stability during load transference from the trailing limb to the leading limb. (A2) During State 3, ankle stiffness increased as downslope angle grew less steep and the upslopes angle grew more steep. Here, the higher stiffness helps store more potential energy, resulting in higher push-off work. (A3) Ankle damping was found to be higher in downslope walking during State 1–2. The higher damping helps counter the higher heel-strike impact. (A4) The ankle reference angle during State 1 and State 4 was close to 0° during level and downslope walking, while it was dorsiflexed to match the slope angle during upslope walking. (A5) In State 2-3, the ankle reference angle greatly influences the generated push-off work. The angle is mildly plantarflexed during State 2, followed by a higher plantarflexed angle in State 3. The steepness of the reference angles increased with the steepness of the slope angle. The values of the angles have been reported in Table 1.

**Table 1:** Ankle and knee reference angles that resulted from solving the optimization problem and post tuning.

	**From optimization**				**Post tuning**			
	**Ankle reference angles (deg)**							
**Slope**	**State 1**	**State 2**	**State 3**	**State 4**	**State 1**	**State 2**	**State 3**	**State 4**
−10.0°	−0.03	−3.94	−5.56	3.58	0.00	2.50	−5.00	0.00
−5.0°	−2.45	−5.30	−14.59	2.75	0.00	0.50	−7.50	0.00
0°	5.60	−11.06	−16.00	0.84	0.00	−2.00	−10.00	2.00
+5°	4.82	−14.78	−16.00	0.75	4.00	−2.00	−10.00	4.00
+10.0°	7.19	−15.0	−16.00	6.37	8.00	−2.00	−10.00	8.00
	**Knee reference angles (deg)**							
**Slope**	**State 1**	**State 2**	**State 3**		**State 1**	**State 2**	**State 3**	
−10.0°	8.90	10.36	30.00		11.97	10.26	16.33	
−5.0°	13.32	14.21	26.00		11.12	8.04	13.86	
0°	10.26	5.83	13.86		10.26	8.00	13.86	
+5.0°	23.52	15.80	20.17		11.12	8.04	13.85	
+10.0°	36.00	24.61	20.00		11.97	10.26	13.85	

*Values for the slope angles not included can be found through linear interpolation*.

The following points are some of the key trends observed in the knee joint parameter functions. (K1) The knee stiffness during State 1–2 was higher at steeper downslope angles, aiding again in countering heel-strike impact and load-transference. (K2) On upslope terrain, the knee stiffness obeyed an opposite trend during State 1–2. The decrease in knee stiffness with the steepness in the upslope angle is believed to enable the required higher knee flexion for terrain adaptation. (K3) During State 3, the knee stiffness is higher on steeper upslope angles allowing for more propulsive knee extension while climbing up. (K4) Knee damping was found to be high during State 2 at steeper slopes (upslope or downslope), while remaining relatively the same during less steep slopes. (K4) The knee reference angles were more flexed on steeper slopes (downslope and Supslope).

**Figure 2:**
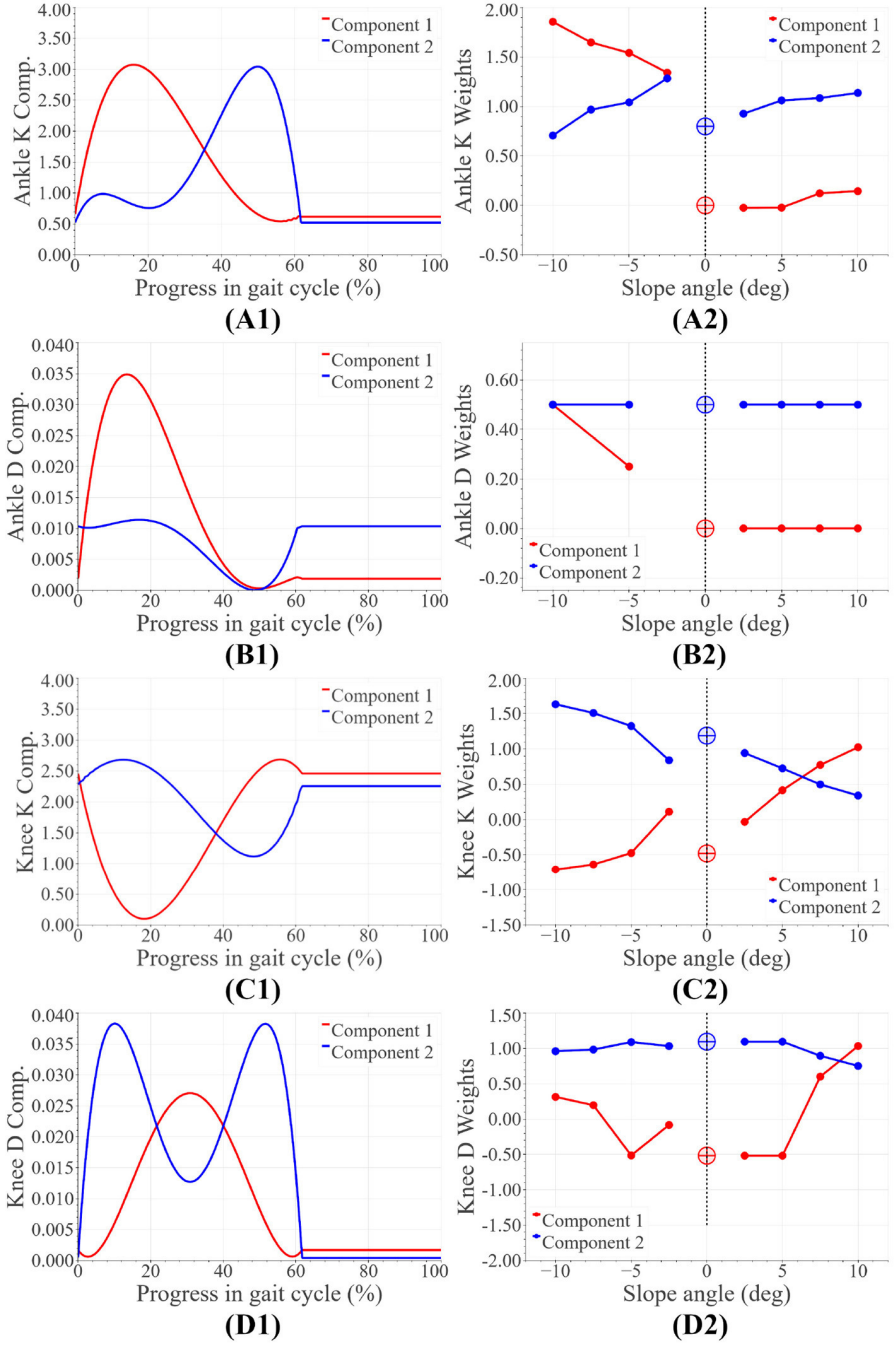
Basis joint parameter functions: panels **(A1,B1)** represent the ankle stiffness (Nm/rad/kg) and damping (Nm/rad/kg) basis functions, while panels **(A2,B2)** are the corresponding weights. Panels **(C1,D1)** represent the knee stiffness (Nm/rad/kg) and damping (Nms/rad/kg) basis functions, while panels **(C2,D2)** are the corresponding weights.

Basis functions spanning all stiffness and damping functions for each joint were extracted using Principal Component Analysis. The functions and their weights have been shown in Figure 2. The entire set of stiffness and damping functions can be found in Supplementary Figure 2. The weights of the basis functions were found to vary monotonically within downslope and upslope walking. Some aberrations were observed, namely: (i) the ankle stiffness weights were higher than anticipated during downslope walking, leading to a discontinuity in weights from downslope to level walking. (ii) the ankle damping weights during downslope walking did not portray strong monotonicity. (ii) the weights corresponding to the knee's functions at −2.5° did not abide by the monotonic trends. We attribute these observations to the anomalies in the data set (discussed in section 2.2). We account for these peculiarities during controller implementation and tuning. The corrective measures are reported in the sections that follow.

## 4. Implementation

**Figure 3:**
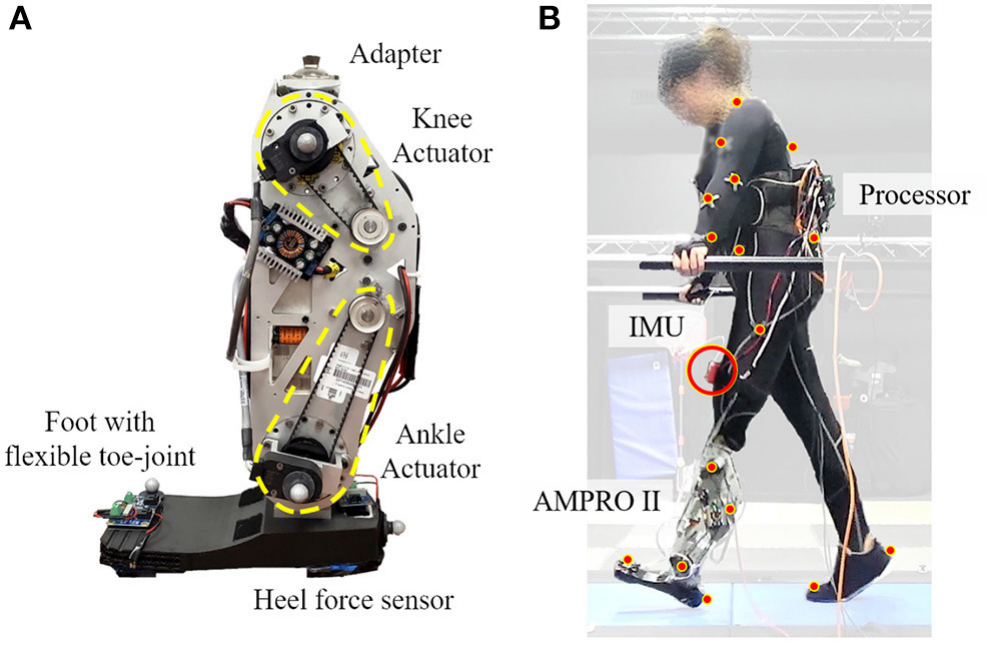
Experimental set up: panel **(A)** is the powered transfemoral prosthesis, AMPRO II, panel **(B)** shows the amputee walking with AMPRO II in a motion capture environment.

The proposed controller was tested on a powered transfemoral prosthesis, AMPRO II (shown in Figure 3A). The following subsections present details on the hardware, controller implementation, and the experiment with an amputee and an able-bodied subject.

### 4.1. Hardware

AMPRO II is operated by an embedded system (BeagleBone Black, element14, Leeds, United Kingdom) that controls an actuated ankle and knee joint. The prosthesis is equipped with a 3D printed foot with a toe joint. A force sensor (FlexiForce A502, Tekscan, South Boston, MA) placed under the heel helps detect heel-strike, while an Inertial Measurement Unit (MPU 9150, SparkFun Electronics, Niwot, CO) affixed to the user's thigh measures the thigh angle. These two parameters help determine the state in the finite state machine and the progress within each state.

### 4.2. State Estimation

The progress in the gait cycle (*t*) is identified using a phase variable that monotonically increases from 0 to 1 as the gait progresses from 0 to 100%. The variable is initialized upon heel-strike detection. A phase portrait of the thigh angle against its integral over the course of gait cycle presents an ellipse. The arc-tangent of the two plotted parameters is among the most successful and popular candidates for a phase variable (Villarreal and Gregg, 2016). Normalizing factors determined in real-time from prior gait cycles, help manipulate the usual elliptical phase portrait into a more circular one. Doing so results in a more linearly varying phase variable and consistent state estimation (Hong et al., 2021).

### 4.3. Controller Tuning

Given the slope's angle, an initial guess for joint stiffness and damping can be found using the impedance basis functions and their weights. The resulting stiffness and damping functions can be tuned further to generate the desired gait kinematics and kinetics. Prior to tuning, both joint parameter functions should be multiplied by the subject's body mass. This study proposes tuning the joint parameter functions as follows.


(9) $$\begin{array}{l} K_{tuned}\left(t\right)=\alpha K\left(t\right)+\gamma \end{array}$$

(10) $$\begin{array}{l} D_{tuned}\left(t\right)=\beta D\left(t\right) \end{array}$$

where α and β are scaling factors, and γ is an offset. Each joint has its own scaling and offset terms. Enumerated below is the tuning procedure. This study recommends tuning the controller for level, −10°, and +10° slope, followed by linearly interpolating parameters for other slope angles.

1. The factor α affects the amount of resistance provided by the system to ankle dorsiflexion and knee flexion. With the ankle, lowering α reduces push-off assistance, while with the knee, lowering α challenges the stability of a flexed knee. Perform the following in iterations.
  (a) Decrease α until the desired ankle dorsiflexion and knee flexion is observed in State 2. This study targeted 5° of ankle dorsiflexion and 10° of knee flexion.
  (b) According to the participant's preference, increase or decrease push-off assistance by, respectively, increasing or decreasing the ankle's plantarflexed reference angle during State 3.
2. Tune β to reach a compromise between the amount of damping preferred by the participant at heel-strike and smooth terrain adaptation post heel-strike.
3. Increase the offset γ to counter gravity and maintain ankle dorsiflexion during swing phase and knee flexion during terminal stance phase.
4. For downslope walking:
  (a) Set the ankle's swing reference angle to 0°.
  (b) Reduce the knee's reference angles to within the acceleration limits of the actuators while maintaining more flexion than level walking. The reference angle during State 2 ensures smooth transition from State 1 to State 3.
5. For upslope walking:
  (a) Increase ankle dorsiflexion and knee flexion in State 1 to facilitate terrain adaptation while respecting the actuators' acceleration limits.
  (b) Set the ankle's swing reference angle to be equal to that in State 1.
  (c) Reduce the knee's reference angle during State 2 to be lower than that in State 1. Accordingly reduce State 3 reference angle to obey the actuators' acceleration limits.
6. Tune the ankle's State 2 reference angle to allow easy transitioning from State 1 to State 3.

### 4.4. Experiment

An indoor experiment was conducted with a transfemoral amputee (female, 164 cm, 66 kg w/o prosthesis). She utilizes a microprocessor knee, X3 Knee (Ottobock), with a Freedom Runaway Foot (Ottobock). Figure 3 depicts the amputee walking with Ampro II. The amputee found walking on slopes uncomfortable even with the accustomed microprocessor prosthesis. Thus the amputee was only asked to walk on slopes angles −5°, +5° with both AMPRO II and her microprocessor knee. The amputee underwent 8 training sessions with AMPRO II before data collection. To demonstrate feasibility of the controller on steeper slopes, a healthy young subject (female, 164 cm, 50 kg) was asked to walk with the prosthesis used a L-shape simulator. The healthy subject walked at −10°, −5°, 0°, +5°, and +10°. All trials were conducted on an AMTI force-sensing tandem treadmill in a motion capture facility with Vicon Vantage motion capture cameras. The amputee chose to walk at 0.54 m/s on slopes, while the able-bodied subject walked at 0.62 m/s. A low speed was selected to avoid fatigue and assure safety. The chosen walking speed was fixed across all slope conditions. The controller was also tested with the amputee at 0.72 m/s on level ground to demonstrate the feasibility of the proposed controller at different walking speeds. The safety of the participant was assured with handrails located on either side of the treadmill. More images of the experiment can be found in Supplementary Figures 3, 4. The experiment protocol has been approved by the Institutional Review Board (IRB) at Texas A&M University (IRB2015-0607F).

To assess the amputee's gait dynamics with the microprocessor knee markers were places on the lower body bony landmarks. Vicon Nexus was used to capture, filter, and interpolate marker data. Visual 3D software was then used to create a model specific to the user and calculate angles and torques.

## 5. Results and Discussion

**Figure 4:**
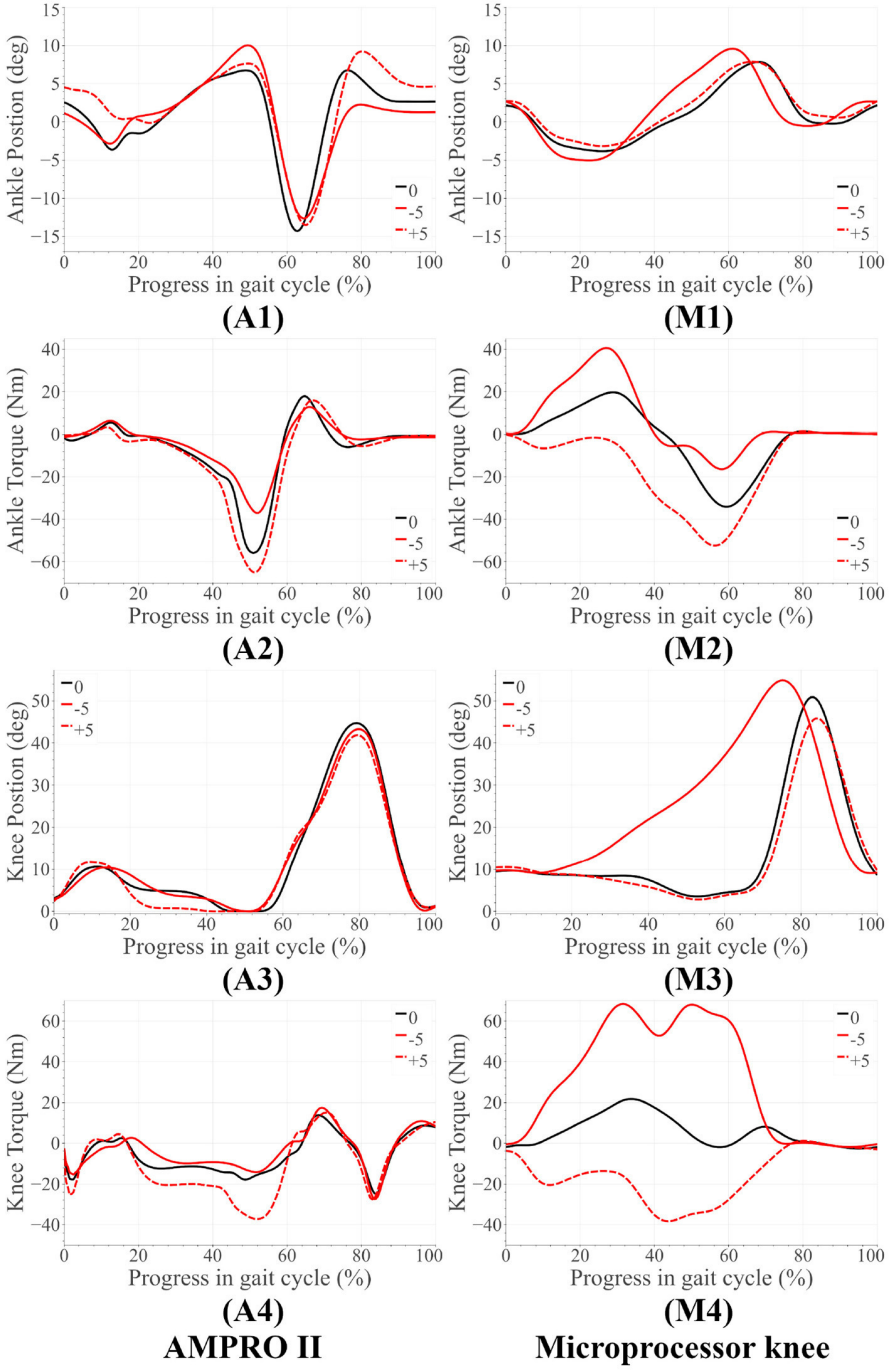
Amputee results for upslope walking and downslope walking. The subfigures labeled **(A)** correspond to the AMPRO II ankle joint, **(M)** are for the Microprocessor knee prosthesis.

**Figure 5:**
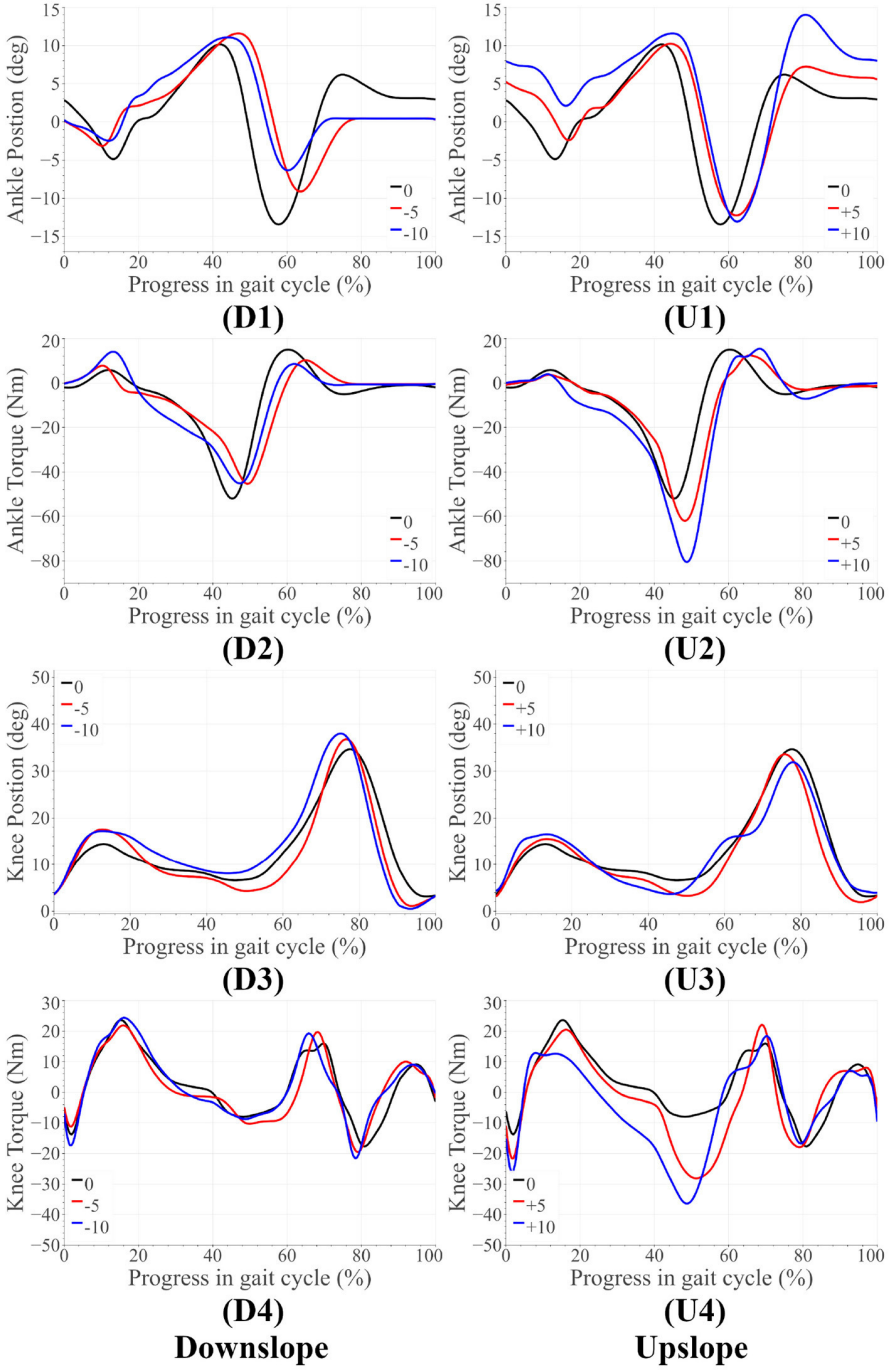
Able-bodied subject results for upslope walking and downslope walking. The subfigures labeled **(U)** correspond to the upslope walking, while those labeled **(D)** are for downslope walking.

**Figure 6:**
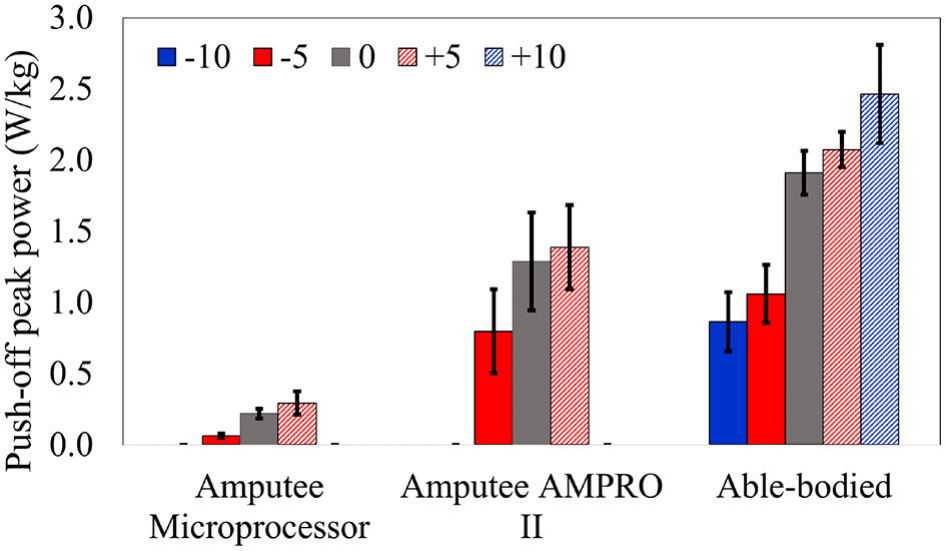
Peak ankle push-off power experienced by the amputee with the microprocessor knee and AMPRO II. Also shown is the peak push-off power experienced by the able-bodied subject with AMPRO II.

**Figure 7:**
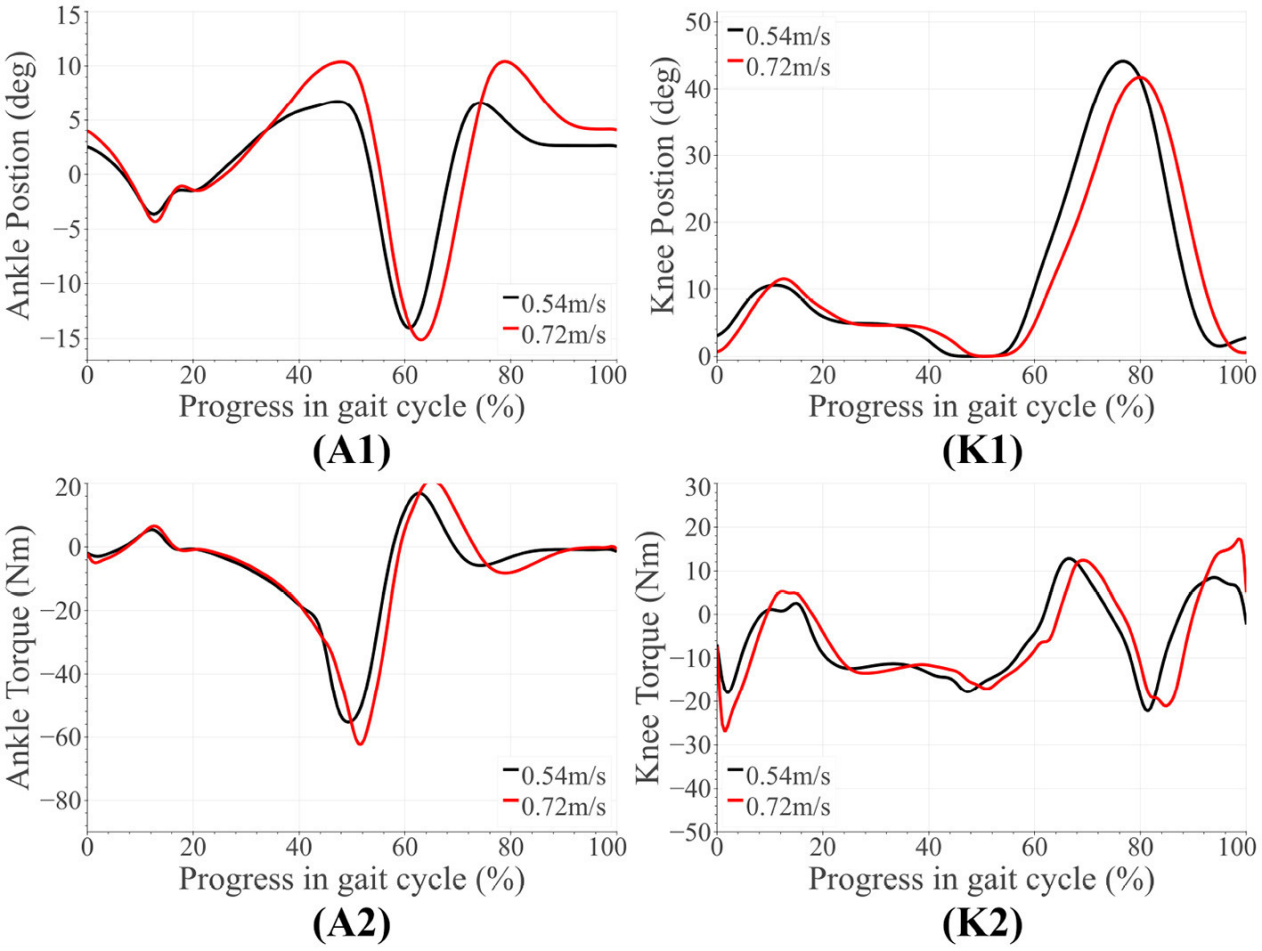
Amputee results for level walking with AMPRO II at different speeds. The subfigures labeled **(A1,A2)** correspond to the ankle, while those labeled **(K1,K2)** are for the knee.

For both amputee and able-bodied subject, the ankle's and knee's tuning parameters were as follows. α = 1, β = 1, and γ = 50 for level and upslope walking. During downslope walking, α = 0.67. This value is consistent with our observation in section 2.1, i.e., the downslope walking kinematic data in Embry et al. (2018a) is higher than the expected value by a factor of 1.5 = 1/α. The tuned reference angles can be found in Table 1. The final proposed scheme in section 6 accounts for this corrective factor. The results for the amputee have been presented in Figures 4, **7**, while those for the able-bodied subject can be found in Figure 5. The gathered kinematics and kinetics were filtered using a Butterworth filter with a cut-off frequency of 20 Hz. The results correspond to the average of 10 gait cycles. Figure 6 reports the peak ankle push-off for both subjects.

### 5.1. Amputee Trials

Figure 7 presents the amputee's walking data with AMPRO II at two speeds. As the walking speed increased, we noted an increase in ankle dorsiflexion during terminal stance phase and ankle plantarflexion during toe-off. The amputee's gait with both AMPRO II and the microprocessor knee on slopes (Figure 4) portrayed some trends similar to those found in healthy walking (see section 2). The ankle push-off moment, amount of knee extension moment between 40 and 60% of the gait cycle, and peak ankle push-off power increased as the slope varied from downslope to upslope. Also observed was higher ankle dorsiflexion at the beginning and end of the gait cycle during upslope walking. During downslope walking, the amputee's microprocessor knee was heavily flexed during stance phase, resulting in high knee flexion moment.

The amputee was able to walk with AMPRO II at various walking speeds. The push-off assistance and the kinematic differences noted earlier have been well-documented in able-bodied walking studies (Embry et al., 2018a; Montgomery and Grabowski, 2018). This proves the feasibility of the control scheme at different walking speeds. While using AMPRO II, the amputee's sloped walking kinematics and kinetics obeyed the monotonic trends found in healthy walking. With more gait training, these results are expected to improve. While using the microprocessor knee on downslopes, knee flexion gradually increased from heel-strike to approximately 70% of the gait cycle (Figure 4M3). This gradual yielding is due to the passive nature of the device, i.e., the device offers no active resistance to knee flexion. Studies such as Alexander et al. (2017) have made similar observations with other microprocessor knees. Additionally, the higher ankle dorsiflexion at the beginning and end of the gait cycle while walking upslope implies terrain adaptation. These results prove the feasibility of the control scheme for amputees.

### 5.2. Able-Bodied Trials

Some notable trends observed in ankle kinematics include: (i) higher dorsiflexed ankle at the beginning and end of the gait cycle on upslopes with the dorsiflexion increasing as the steepness of the slope increased, (ii) lesser toe-off plantarflexion on downslopes, (iii) higher knee flexion during initial stance phase on sloped terrain than level ground. In terms of kinetics, we observed: (i) that the ankle peak torque and power (Figure 7) varied monotonically with the angle as it varied from −10° to +10°, (ii) higher knee extension torque on upslopes.

The variation in ankle angle at the beginning and end of the gait cycle facilitates terrain adaptation. The higher plantarflexion at toe-off during upslope walking is correlated to the higher push-off torque and power. Higher push-off assistance is required as the slope varies −10° to +10°. The higher extension torque, ankle push-off torque and power on upsloped terrain are all correlated with this need for higher push-off assistance. All of these trends are observed in healthy walking (detailed in section 2), proving the feasibility of the control scheme on steeper slopes.

### 5.3. Comparison Against the State-of-the-Art

As mentioned in section 1.1, other attempts at sloped walking with impedance control strategies include (Sup et al., 2011; Fey et al., 2014; Bhakta et al., 2019). In this section, we will compare our results against the cited works using three metrics: number of tuning parameters per joint, capability of terrain adaptation, and variation in push-off assistance with the slope angle. Sup et al. (2011) had 15 tuning parameters per joint, of which 8–12 parameters were manually tuned for each tested slope condition (0°, +5°, +10°). The results indicated terrain adaptation and increase in push-off power as the slope angle increased. Both Fey et al. (2014) and Bhakta et al. (2019) implemented control strategies wherein the parameters varied as linear functions of the instantaneous joint angle or shank force. Overall, there were at least 12 tuning parameters per joint. Fey et al. (2014) tested the strategy at 0° and +10°. The results showed no sign of terrain adaptation, however the push-off torque increased from level to inclined walking. Bhakta et al. (2019), on the other hand, tested the control strategy on various up and downslope walking conditions: 0°, ±7.8°, ±11.0°, ±12.4°, ±14.0°). The kinematic results showed some signs of terrain adaptation from level to sloped walking, but there was no identifiable difference from one slope angle to another within downslope or upslope walking results. Moreover, Bhakta et al. (2019) does not present kinetic results, limiting our ability to gauge the controller's performance.

Of all prior listed works, Sup et al. (2011) is the only study that successfully accomplished terrain adaptation and slope-based power assistance scaling during upslope walking. Our controller accomplishes the same with far fewer tuning parameters per joint (7–8 parameters per joint) than all three listed works. Unlike (Sup et al., 2011; Fey et al., 2014), our controller was tested on both up and downslope walking conditions, further strengthening our controller's performance. Further, unlike (Bhakta et al., 2019) which is limited to kinematic analysis, our controller can reproduce both kinematic and kinetic trends of healthy human sloped walking. Said trend reproduction is observable not only from downslope to upslope walking, but also from one slope angle to another within both downslope and upslope walking. Thus, our controller is a significant improvement on existing sloped walking impedance control strategies.

## 6. Conclusion

We propose a sloped walking control framework with fewer tuning parameters than the state-of-the-art controllers. The framework includes impedance control during stance phase and trajectory tracking during swing phase. The smooth transition between the two is facilitated by Bezier curves. The joint control parameters were determined through a data-driven optimization. Basis functions spanning the entire set of joint parameter functions were found through Principle Component Analysis. Given any slope angle, the stiffness and damping control parameters can be found as follows:


(11) $$\begin{array}{l} K_{tuned}\left(t\right)=\alpha \left(w_{K1}\left(\psi \right)K_{Comp1}\left(t\right)+w_{K2}\left(\psi \right)K_{Comp2}\left(t\right)\right)+\gamma \end{array}$$

(12) $$\begin{array}{l} D_{tuned}\left(t\right)=\beta \left(w_{D1}\left(\psi \right)D_{Comp1}\left(t\right)+w_{D2}\left(\psi \right)D_{Comp2}\left(t\right)\right) \end{array}$$

where *K*_*Comp*1_, *K*_*Comp*2_ represent stiffness basis functions, while *D*_*Comp*1_, *D*_*Comp*2_ are the damping basis functions. The associated polynomial coefficients can be found in Table 2. The weights for these basis polynomials vary as functions of the slope angle and are represented by *w*_*K*1_(ψ), *w*_*K*2_(ψ), *w*_*D*1_(ψ) and *w*_*D*2_(ψ). The coefficients of the weights have been tabulated in Table 3. A thorough tuning routine has also been prescribed in this paper. The tuning process can be automated using rule-based fuzzy logic. Testing with an amputee and able-bodied subject proved the feasibility of the proposed scheme at varying slope angles. Monotonic trends consistent with healthy human walking data were observed in both kinematics and kinetics. To name a few: push-off assistance (from both ankle and knee joint) increased as the slope angle increased from downslope angles to upslope angles, and the ankle angle at the beginning and end of the gait cycle varied according to the slope angle–enabling terrain adaptation.

**Table 2:** The coefficients of the implemented stiffness and damping polynomials.

**Comp**.	***k*_4_**	***k*_3_**	***k*_2_**	***k*_1_**	***k*_0_**
**Ankle stiffness (Nm/rad/kg)**					
Comp. 1	−108.61	234.61	−160.63	35.23	0.66
Comp. 2	−476.16	493.56	−146.91	14.63	0.52
**Knee stiffness (Nm/rad/kg)**					
Comp. 1	−13.291	−74.669	96.030	−27.672	2.525
Comp. 2	77.418	−41.999	−8.480	2.949	2.317
**Comp**.	***d*_4_**	***d*_3_**	***d*_2_**	***d*_1_**	***d*_0_**
**Ankle damping (Nms/rad/kg)**					
Comp. 1	−3.41	5.75	−3.18	0.58	0.00
Comp. 2	1.75	−1.60	0.36	−0.02	0.01
**Knee damping (Nms/rad/kg)**					
Comp. 1	3.905	−4.844	1.622	−0.074	0.001
Comp. 2	−13.022	16.146	−6.402	0.866	0.000

*The word Component has been abbreviated to Comp*.

**Table 3:** Weight functions for the ankle and knee joint control parameter basis functions.

	**Ankle**	**Knee**
*w*_*K*1_(ψ)	−0.137ψ − 0.060 for ψ <0, 0 otherwise	0.005ψ^2^ + 0.090ψ − 0.270
*w*_*K*2_(ψ)	0.032ψ + 0.84	−0.001ψ^2^ − 0.065ψ + 1.106
*w*_*D*1_(ψ)	−0.05ψ for ψ <0, 0 otherwise	0.001ψ^3^ + 0.014ψ^2^ − 0.002ψ − 0.621
*w*_*D*2_(ψ)	0.5	−0.003ψ^2^ − 0.007ψ + 1.118

Future work involves improving the phase variable based estimation scheme for sloped walking. Currently, phase variable schemes do not account the relationship between toe-off timing and slope angle (i.e., toe-off timing is delayed as the slope varies from steep downslope to steep upslope terrain). Improving the scheme would greatly reduce the standard deviations of peak push-off power seen in Figure 6. A possible approach is to mount a force sensor at the toe and update the toe-off timing–in the finite state machine–from one gait cycle to another. Another improvement to the existing control scheme involves employing a continuously varying reference angle. Doing so would improve the stability of the system under uncertainties in state estimation (Mohammadi and Gregg, 2019). Additionally, a continuously varying reference angle could further reduce the number of states in the finite state machine, further easing user customization of the proposed control scheme.

## Data Availability Statement

The raw data supporting the conclusions of this article will be made available by the authors, without undue reservation.

## Ethics Statement

The studies involving human participants were reviewed and approved by Institutional Review Board at Texas A&M University. The patients/participants provided their written informed consent to participate in this study.

## Author Contributions

NA was the primary contributor for the proposed control scheme, data collection and processing. SP contributed to data collection and analysis. WH assisted with implementation of the control scheme. PH served as the principal investigator. All authors contributed to writing and reviewing this paper.

## Conflict of Interest

The authors declare that the research was conducted in the absence of any commercial or financial relationships that could be construed as a potential conflict of interest.

## Publisher's Note

All claims expressed in this article are solely those of the authors and do not necessarily represent those of their affiliated organizations, or those of the publisher, the editors and the reviewers. Any product that may be evaluated in this article, or claim that may be made by its manufacturer, is not guaranteed or endorsed by the publisher.
