# Supplementary material for: Control Framework for Sloped Walking With a Powered Transfemoral Prosthesis
Source: Front Neurorobot. 2022 Jan 11;15:790060. doi: 10.3389/fnbot.2021.790060 (PMC8786733; doi:10.3389/fnbot.2021.790060)
Supplement: Supplementary file 1 [file Data_Sheet_1.PDF]

Supplementary Material

1 SUPPLEMENTARY TABLES AND FIGURES

1.1 Figures

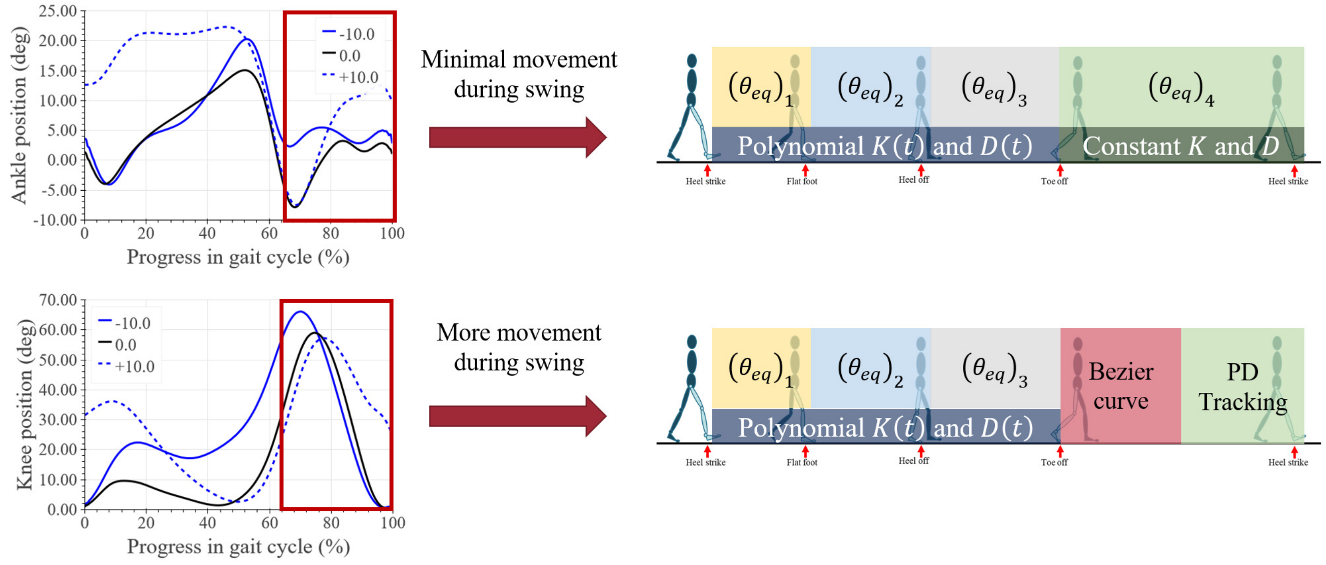

Figure S1. Control framework for sloped walking.

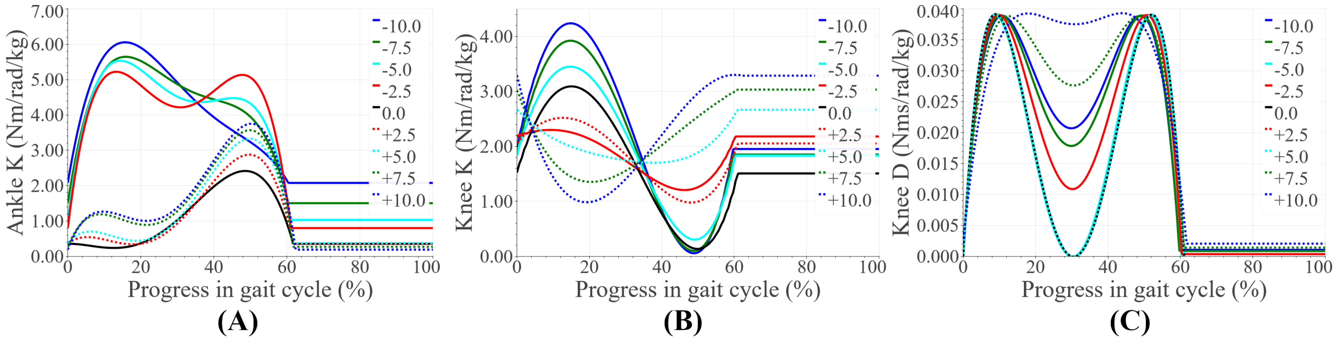

Figure S2. Joint parameter functions: **(A)** Ankle stiffness, **(B)** Knee stiffness, **(C)** Knee damping

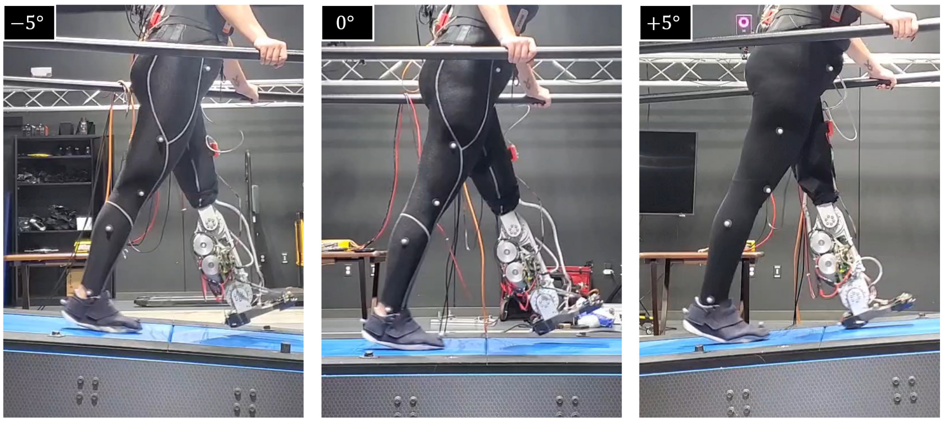

Figure S3. Amputee walking with Ampro II on three different terrain conditions

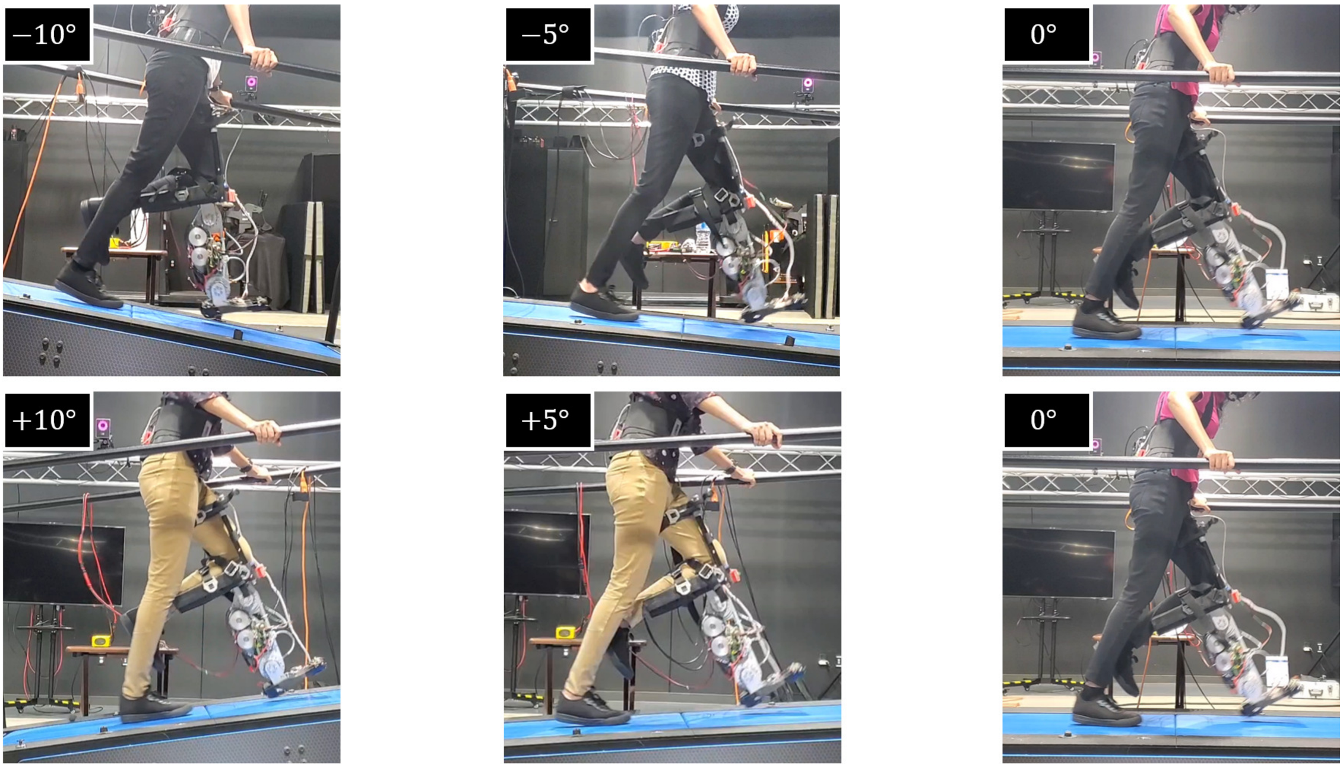

Figure S4. Able-bodied emulated prosthetic walking on five different terrain conditions
